# Medical ozone alleviates acute lung injury by enhancing phagocytosis targeting NETs *via* AMPK/SR-A1 axis

**DOI:** 10.7555/JBR.38.20240038

**Published:** 2024-05-29

**Authors:** Chenxiao Yan, Yong Zhang, Lai Jin, Xiaojie Liu, Xuexian Zhu, Qifeng Li, Yu Wang, Liang Hu, Xueming He, Hongguang Bao, Xia Zhu, Qian Wang, Wen-Tao Liu

**Affiliations:** 1 The Key Laboratory of Rare Metabolic Disease, Department of Biochemistry and Molecular Biology, the Key Laboratory of Human Functional Genomics of Jiangsu Province, Key Laboratory of Targeted Intervention of Cardiovascular Disease, Collaborative Innovation Center for Cardiovascular Disease Translational Medicine, Nanjing Medical University, Nanjing, Jiangsu 211166, China; 2 Department of Anesthesiology, Nanjing First Hospital, Nanjing Medical University, Nanjing, Jiangsu 210006, China; 3 Jiangsu Key Laboratory of Neurodegeneration, Department of Pharmacology, Nanjing Medical University, Nanjing, Jiangsu 211166, China; 4 Department of Anesthesiology, the Affiliated Drum Tower Hospital, Medical School of Nanjing University, Nanjing, Jiangsu 210009, China; 5 Center for Clinical Research and Translational Medicine, Department of Anesthesiology, the Affiliated Lianyungang Oriental Hospital of Xuzhou Medical University, Lianyungang, Jiangsu 222042, China; 6 Department of Anesthesiology, the Affiliated Lianyungang Oriental Hospital of Kangda College of Nanjing Medical University, Lianyungang, Jiangsu 222042, China

**Keywords:** SR-A1, NETs, ALI, phagocytosis, ozone therapy

## Abstract

Acute lung injury (ALI) linked to sepsis has a high mortality rate, with limited treatment options available. In recent studies, medical ozone has shown the potential to alleviate inflammation and infection. Here, we aimed to evaluate therapeutic potential of medical ozone in a mouse model of the sepsis-induced ALI by measuring behavioral assessments, lung function, and blood flow. Protein levels were quantified by Western blotting. *In vitro*, we performed experiments on bone marrow-derived macrophages (BMDMs) to investigate the effect of adenosine monophosphate (AMP)-activated protein kinase (AMPK) inhibitors and agonists on their phagocytic activity. The results showed that medical ozone significantly improved the survival rate, ameliorated lung injury, and enhanced lung function and limb microcirculation in mice with ALI. Notably, medical ozone inhibited the formation of neutrophil extracellular traps (NETs), a crucial factor in the ALI development. Additionally, medical ozone counteracted the elevated levels of tissue factor, matrix metalloproteinase-9, and interleukin-1β. In the ALI mice, the effects of ozone were abolished, and BMDMs showed an impaired capacity to engulf NETs following the *Sr-a1* knockout. Under normal physiological conditions, the administration of an AMPK antagonist showed similar effects on the *Sr-a1* knockout, significantly inhibiting the phagocytosis of NETs by BMDMs. In contrast, AMPK agonists enhanced this phagocytic process. In conclusion, medical ozone may alleviate the sepsis-induced lung injury through the AMPK/SR-A1 pathway, thereby enhancing the phagocytosis of NETs by macrophages.

## Introduction

Defined as "life-threatening organ dysfunction resulting from a dysregulated host response to infection" ^[ 1] ^, sepsis remains a prominent cause of mortality worldwide, particularly in the context of the ongoing novel coronavirus pneumonia pandemic. The lung is the most common site of infection leading to sepsis ^[ 2– 4] ^, and is particularly vulnerable during sepsis, with more than 50% of sepsis patients developing acute lung injury (ALI) or acute respiratory distress syndrome (ARDS) ^[ 5– 6] ^. Given the rise in antibiotic resistance, the controversial use of glucocorticoids, and the limited availability of alternative therapies, there is a pressing need in current clinical practice to identify safer and more efficacious treatments for sepsis ^[ 7] ^.

The activation and infiltration of neutrophils have been identified as pivotal events in ALI ^[ 8– 9] ^. Neutrophil recruitment plays a crucial role in the body's immune response against pathogens, and the formation of neutrophil extracellular traps (NETs) during this process aids in pathogen capture and elimination ^[ 10– 11] ^. NETs form a unique three-dimensional mesh structure with DNA as the backbone, which efficiently adheres to and captures pathogens, while its inter-backbone contains bactericidal and permeability-increasing proteins, such as citrullinated histone H3 (H3Cit), neutrophil elastase (NE), and myeloperoxidase (MPO), and these proteins facilitate the elimination of pathogens. However, some emerging evidence suggests that NETs may also trigger noninfectious inflammation and contribute to autoimmune diseases ^[ 12– 13] ^. In the context of septicemic lung injury, NETs enriched with tissue factor (TF) and activated platelets may induce immune thrombosis, exacerbating disease progression ^[ 14] ^. Notably, anticoagulants, such as heparin and tissue factor pathway inhibitors (TFPI), have demonstrated some therapeutic potential in ameliorating ARDS and ALI ^[ 15] ^. In the mouse model of sepsis, NETs were predominantly observed in the lungs, promoting fibrin deposition and lung injury. The inhibition of CXCR1/2 has been reported to reduce NET formation, mitigate organ damage, and improve survival rates, without compromising pathogen clearance ^[ 16] ^. Overall, targeted reduction of excessive NET accumulation represents a promising therapeutic strategy for the management of lung injury.

Scavenger receptors (SRs) comprise a diverse family of membrane receptors categorized into twelve classes (A to L) based on their structure and function ^[ 17] ^. These receptors play a crucial role in the recognition and clearance of some modified self-targets or non-self-targets through processes such as phagocytosis, endocytosis, and adhesion ^[ 18] ^. Pathophysiological processes associated with SRs primarily involve cardiovascular diseases, pathogenic infections, and immune surveillance ^[ 19] ^. Although functional investigations of SRs focus on atherosclerosis, their involvement in host immune defense is garnering an increasing attention ^[ 20– 21] ^. SR-A1, mainly expressed in macrophages, dendritic cells, and certain endothelial cells, acts as a pattern recognition receptor in synergy with Toll-like receptor 4 (TLR4). In the presence of lipopolysaccharide (LPS), SR-A1 stimulates NF-κB activation and the release of inflammatory factors in macrophages ^[ 22] ^. Recent studies have uncovered a potential role for SR-A1 in promoting an anti-inflammatory response by fostering the M2 macrophage phenotype in cardiovascular disease ^[ 23] ^. Macrophages have been reported to degrade NETs through pre-digestion and subsequent uptake ^[ 24] ^. The formation of NETs is significantly elevated in ARDS patients, and the activation of adenosine monophosphate (AMP)-activated protein kinase (AMPK) using metformin has been shown to enhance phagocytosis and clearance of NETs by macrophages ^[ 25] ^.

The therapeutic potential of medical ozone/oxygen mixtures, hereafter referred to as ozone, has garnered attention because of its potent ability to regulate and mitigate oxidative stress at precise therapeutic doses. Investigators have proposed ozone as a potentially effective and cost-controlled strategy for managing chronic inflammatory diseases, including chronic obstructive pulmonary disease, asthma, bronchiectasis, and pulmonary fibrosis ^[ 26– 27] ^. Ozone exerts its effects by activating and modulating the immune system, leading to anti-inflammatory and antioxidant responses ^[ 28– 29] ^. However, the exact mechanisms underlying ozone therapy are still not fully understood. Our previous study demonstrated that ozone therapy might activate AMPK and ameliorate chemotherapeutic enteritis ^[ 30] ^. Building upon this work, our present study investigated the potential and underlying mechanisms of ozone to attenuate septic lung injury.

## Materials and methods

### Animals and treatment

Male C57BL/6J mice, 6–8 weeks old, weighing 22.5 (± 2.5) g, were purchased from Charles River Laboratories (Beijing, China), and *Sr-a1* ^*−/−*^ mice on a C57BL/6J background were kindly provided by Professor Qi Chen (Nanjing Medical University). All experiments involving animals were conducted as approved by the Animal Ethical and Welfare Committee of Nanjing Medical University (Permit No. IACUC-2305042) and carried out following the established guidelines for the care and use of animals. The mice were housed in a controlled environment at 22 (± 2) ℃ with a 12 h light-dark cycle (lights on at 8:00 a.m.) and had access to food and water *ad* *libitum*. The mice were used after 5 days of adaptation to the environment. For each group of experiments, the mice were matched by age and body weight.

The model of sepsis-induced ALI was established by a single intraperitoneal (i.p.) injection of 12.5 mg/kg LPS (Cat. #L2630, Sigma-Aldrich, St. Louis, MO, USA) in mice. For the survival rate experiments, three concentrations of ozone were used, and 15 μg/mL ozone was selected as the optimal concentration for the rest of the experiments. As a potential therapeutic strategy, the protein arginine deiminase 4 (PAD4) inhibitor Cl-amidine (Cat. #S8141, Selleck, Houston, TX, USA; 10 mg/kg) or ozone (0.5 mL per mouse) was injected (i.p.) into mice 2 h before model establishment. The ozone/oxygen mixture was generated by Medozon compact (Herrmann Apparatebau, Germany). Ozone obtained from medicinal-grade oxygen was injected (i.p.) into mice immediately. Mice were randomly divided into four groups of six each according to the treatment. The groups were: the control group (no treatment), LPS group, ozone + LPS group, and ozone group.

### Non-invasive measurement of lung function in mice

Whole-body plethysmography was used to measure various indexes of lung function in mice. Specifically, mice were labeled with picric acid before detection and then placed in a detector to adapt for 2 min to ensure the room was quiet. This was followed by a continuous monitoring of the mice's breathing for 8 min. The pressure change in the volumetric cavity caused by animal respiration was recorded by a screen respiratory sensor in the cavity and amplified by an amplifier. Based on these pressure changes, we used the analysis software algorithm to deduce the indexes of lung function.

### Left lower limb blood flow measurement

The blood flow of the left lower limbs of the mice was measured by a Laser Speckle Blood Flow Imager (Simopto, Wuhan, China). Specifically, mice sedated with isoflurane had their limbs scanned by a low-power laser beam from a computer-controlled optical scanner, with the scanning head placed parallel to the exposed lower limb at a distance of about 20 cm during this period. After about 10 s, a color-coded image representing a specific relative perfusion level was displayed on the video monitor. Blood flow values were recorded and assessed by the Moor FLPIR View V40 program (Gene & I Scientific Ltd., Beijing, China).

### Histologic analysis

For the histological analysis, the mice were anesthetized with isoflurane and then sacrificed quickly. The lung tissues were collected. The samples were fixed in 4% paraformaldehyde fix solution for 24 h, dehydrated in ethanol, and finally embedded in paraffin. Then, microtome sections (5 μm) were cut and stained with hematoxylin and eosin (H&E). Images were taken under a light microscope (Leica, Germany) by two investigators blinded to the group assignments. Three random fields of each lung section were examined.

### Immunofluorescence assay

For formalin-fixed paraffin-embedded tissues, mounted sections were dewaxed with xylene, rehydrated, and blocked. The sections were then incubated with primary antibodies for histone H3 (1∶200; Cat. #ab5103, Abcam, Cambridge, UK) overnight at 4 ℃. Then, the sections were washed and stained with fluorophore-conjugated secondary antibodies (Alexa Fluor 488-conjugated donkey anti-rabbit, Cat. #711-545-152, Jackson ImmunoResearch, West Grove, PA, USA) at room temperature for 2 h. After washing three times with PBS, the samples were sealed with DAPI Fluoromount-G (Cat. #0100-20, Southern Biotech, Birmingham, AL, USA) and were examined under fluorescence microscopy (LEICA DM2500) for morphological details. The examination was carried out blind.

### Isolation of the bone marrow-derived macrophages (BMDMs)

After euthanasia, the mice were swabbed with alcohol cotton balls, and then the tibia and femur were extracted. A 25-gauge needle and a 10-cc syringe filled with RPMI-1640 (Cat. #KGM31800N-500, Keygen Biotech, Nanjing, China) supplemented with 10% fetal bovine serum (FBS; Cat. #04-001-1ACS, Biological Industries, Kibbutz Galuyot, Israel) were used to flush the bone marrow cells from both ends of the bone shafts. The cell suspension filtered through a 70-μm nylon cell strainer (Cat. #CSS013070, JET BIOFIL, Guangzhou, China) was centrifuged at 1500 *g* for 5 min. DMEM (Cat. #KGM12800N-500, Keygen Biotech) supplemented with 10% (v/v) FBS, 1% penicillin/streptomycin (Cat. #KGY0023, Keygen Biotech), and 10% L929 cell supernatant was used to differentiate and maintain BMDM cells. BMDMs were seeded in confocal dishes for 7 days, stimulated with LPS at 100 ng/mL and IFN-γ at 50 ng/mL for 24 h to induce type M1 standby.

### NETs isolation

As mentioned above, cells from the bone marrow were harvested. The cells were resuspended in 3 mL of sodium chloride physiological solution and then layered on top of 9 mL of Histopaque 1077 (Cat. #10771, Sigma-Aldrich) in a 50-mL centrifuge tube. After centrifugation at 2000 *g* at 4 ℃ for 20 min, the supernatant was discarded. The bottom cell pellet was resuspended in 5 mL of sodium chloride physiological solution and layered on top of 10 mL of Histopaque 1119 (Cat. #11191, Sigma-Aldrich) in a 50-mL centrifuge tube. After centrifugation at 2000 *g* at 4 ℃ for another 20 min, neutrophils were harvested from the cloudy white cell layer at the saline-Histopaque 1119 boundary. During the addition of the cell suspension, the drip rate was kept constant and slow, while the centrifuge tube was tilted to eliminate the effects of gravity, ensuring the separation of high-purity and high-yield neutrophils. The collected neutrophils were washed with RPMI-1640 supplemented with 10% FBS and 1% penicillin/streptomycin twice and centrifuged at 1500 *g* at 4 ℃ for 7 min.

The isolated neutrophils were seeded in a 10-cm Petri dish pre-coated with poly-L-lysine and then stimulated with LPS (1 μg/mL) for 4 h in RPMI-1640. After stimulation, the supernatant was discarded carefully, leaving NETs and neutrophils adhered to the bottom of the Petri dish. Then, 15 mL of cold PBS (without Ca ^2+^ and Mg ^2+^) was used to wash all adherent NETs and neutrophils from the bottom. After centrifugation at 450 *g* for 10 min, neutrophils in the pellet at the bottom were separated from the cell-free NETs in the supernatant. The supernatant was centrifuged at 17 000 *g* at 4 ℃ for 10 min, which allowed NETs to precipitate to the bottom. The supernatant was discarded and the pellets were resuspended in cold PBS. The NETs concentration in the sample was measured by a NanoDrop spectrophotometer (Nano-100, AllSheng, Hangzhou, China) and adjusted to 140–180 ng/μL for subsequent assays.

### Macrophage endocytosis of NETs

NETs were stained with 1 mmol/L Sytox Orange (S34861, ThermoFisher, Waltham, MA, USA) at 4 ℃ for 30 min and then centrifuged at 17000 *g* at 4 ℃ for 5 min. The supernatant was discarded, and the pellets were resuspended in PBS to obtain stained-labeled NETs. LPS (1 μg/mL) was used to stimulate BMDMs for 12 h to induce inflammation. BMDMs were treated in advance with ozone (15 μg/mL, dissolved in an equal volume in the medium) for 2 h, while acadesine (AICAR; 300 μmol/L, HY-13417, MCE, NJ, USA) or Compound C (20 μmol/L, T6146, TargetMol, MA, USA) was used to treat BMDMs for 15 min. Stained-labeled NETs were incubated with BMDMs in the confocal dish for 15 min, then rinsed lightly with ice-cold PBS three times, and photographed under confocal microscopy (LSM 800, Zeiss). The 543 nm spectral line excitation and the white light were merged to reflect macrophage endocytosis of NETs.

### Quantification of the circulating free DNA (cf-DNA)

Mice plasma was collected by centrifugation at 3000 *g* for 5 min. cf-DNA in plasma was quantified according to the manufacturer's instructions of the Quant-iT PicoGreen dsDNA Assay kit (Cat. #P11496, Invitrogen, Carlsbad, CA, USA). Plasma samples were diluted at a ratio of 1∶4, and measured by a microplate reader (Cytation5, Biotek) with standard fluorescein wavelengths (excitation, 480 nm; emission, 520 nm).

### Western blotting assay

Lung tissues were collected and washed with ice-cold PBS before lysing in radioimmunoprecipitation assay (RIPA) lysis buffer containing a phosphatase protease inhibitor and PMSF. Total protein concentrations were quantified by BCA Protein Assay Kit (Cat. #23225, Thermo Fisher), and 25 μg of proteins were loaded, separated by SDS-PAGE, and electrophoretically transferred onto polyvinylidene fluoride membranes. The membranes were blocked with 10% whole milk in TBST (Tris-HCl, NaCl, Tween 20) for 2 h at room temperature, and were probed with primary antibody at 4 ℃ overnight. The primary antibodies were NE (1∶1000; Cat. #ab68672, Abcam), H3 (1∶1000; Cat. #17168-1-AP, Proteintech, Wuhan, China), H3Cit (1∶1000; Cat. #ab281584, Abcam), TF (1∶500; Cat. #sc-374441, Santa Cruz, CA, USA), MMP-9 (1∶1000; Cat. #10375-2-AP, Proteintech), IL-1β (1∶1000; Cat. #AF-401-NA, R&D Systems, CA, USA), and β-actin (1∶50000; Cat. #AC02, Abclonal, Wuhan, China). Then, the membranes were incubated with horseradish peroxidase-coupled secondary antibodies from Sigma. Data were acquired with a Molecular Imager (ChemiDoc, Bio-Rad).

### Statistical analysis

Prism 9.0 (GraphPad) was used to perform all statistical analyses. Image J software was used to quantify the gray value. Adobe Illustrator software was used for mapping. Data were presented as mean ± standard deviation and statistically evaluated by the one-way or two-way ANOVA. All tests of statistical significance were two-sided, and the statistical significance was set at *P* < 0.05.

## Results

### Sepsis-induced microcirculation disturbance and the deterioration of lung function were significantly alleviated by ozone

**Figure 1:**
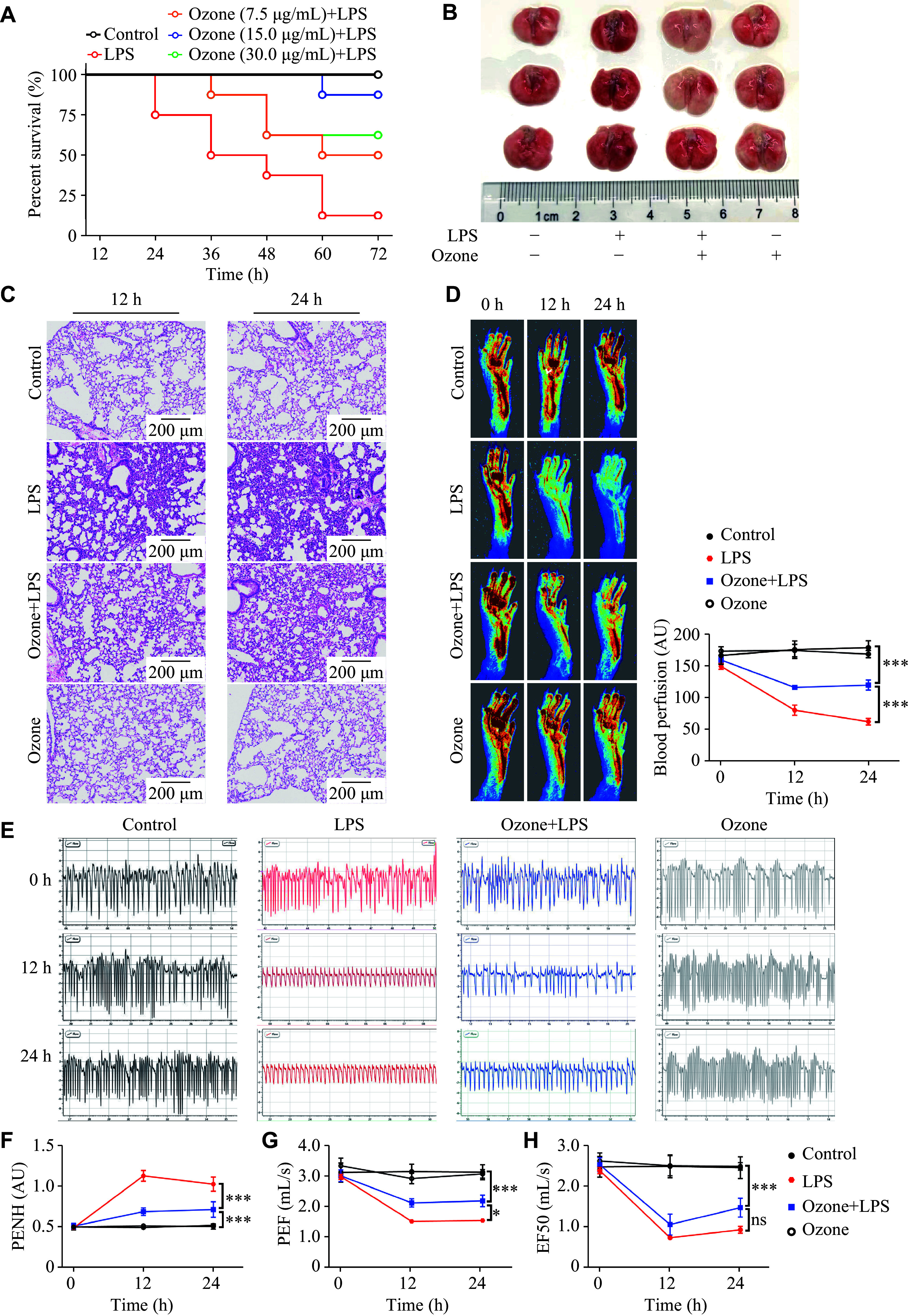
Ozone significantly alleviated the LPS-induced limb microcirculation disturbance and the deterioration of lung function. A: Mice were injected intraperitoneally with LPS (12.5 mg/kg) 2 h after the intraperitoneal injection of 0.5 mL ozone at the indicated concentrations. The survival rate of mice was monitored every 12 h for three consecutive days ( *n* = 8). B–H: Mice were injected intraperitoneally with LPS (12.5 mg/kg) 2 h after the intraperitoneal injection of 0.5 mL ozone (15 μg/mL). Representative photographs of lung tissues 12 h after the LPS injection ( *n* = 3; B). The H&E sections of the lung tissues at 12 h and 24 h after the LPS injection ( *n* = 3; C). Blood flow in the lower limbs of mice was evaluated at 0 h, 12 h, and 24 h after the LPS injection ( *n* = 6; D). Representative eight-second waveforms of lung function at 0 h, 12 h, and 24 h after the LPS injection (E). Indexes of lung function include enhanced pause (PENH), peak expiratory flow (PEF), and mid-expiratory flow (EF50) ( *n* = 6; F–H). Data are presented as mean ± standard deviation. ^***^ *P* < 0.001 by one-way ANOVA followed by Tukey's tests for multiple comparisons. Abbreviations: LPS, lipopolysaccharide; AU, arbitrary unit; ns, not significant.

As shown in ***Fig. 1A***, LPS reduced the survival rate (12.5%) of the mice at 72 h, compared with that of the control group (100%), while pre-administration of different concentrations of ozone rescued the LPS-induced death. Ozone at a concentration of 15 μg/mL achieved the maximal effect, and was therefore used in all subsequent experiments. Upon examination of the collected lungs, the LPS group exhibited signs of bleeding and swelling, whereas ozone treatment effectively alleviated these pathological changes ( ***Fig. 1B***). The histological analysis through H&E staining revealed distinct pathological features in the model group, including focal consolidation, alveolar collapse, alveolar septal edema and widening, red blood cell leakage, and the infiltration of inflammatory cells, predominantly neutrophils. Conversely, ozone treatment protected structural integrity of lung tissues, with only a minimal presence of exudative red blood cells and infiltrated inflammatory cells ( ***Fig. 1C***).

The decreased oxygenation at the fingertip is commonly observed in patients with acute lung injury, acute respiratory distress syndrome, or novel coronavirus pneumonia. Similarly, the model mice in the current study exhibited the peripheral microcirculation disturbance in the limbs. Surprisingly, ozone pretreatment significantly ameliorated the microcirculation disturbance in the limbs following modeling in the mice ( ***Fig. 1D***). Mouse respiration was monitored to further investigate the protective effects of ozone ( ***Fig. 1E***). After a 12-h stimulation with LPS, the lung function of the mice was rapidly affected, leading to a deterioration of airway obstruction and airway conduction parameters. However, ozone preconditioning demonstrated an improvement in lung function by reducing airway obstruction and increasing peak expiratory flow. Notably, ozone treatment did not appear to have any effect on mid-expiratory flow (EF50; ***Fig. 1F***– ***1H***).

### Sepsis-induced ALI was significantly alleviated by ozone

**Figure 2:**
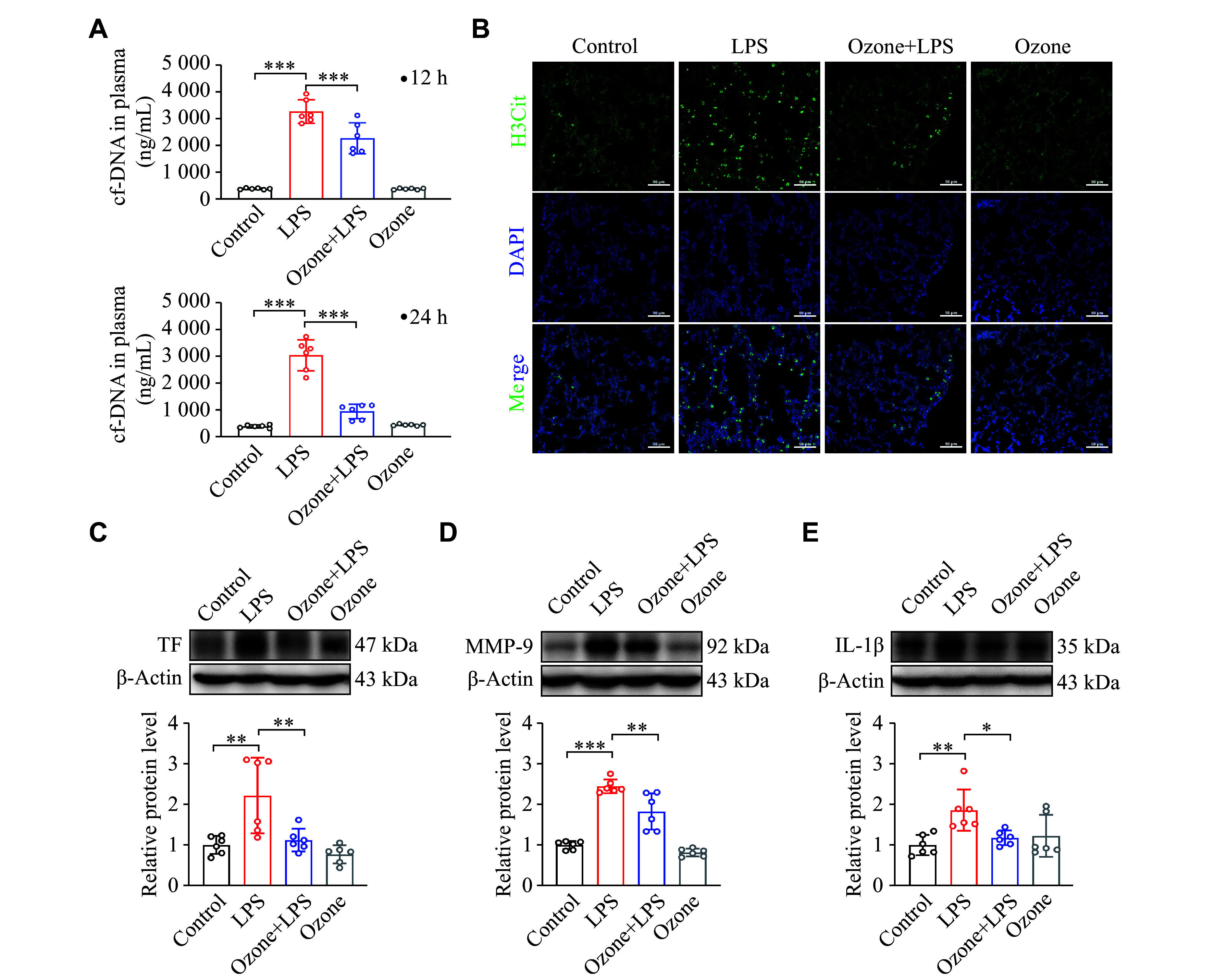
Ozone significantly ameliorated the LPS-induced lung injury. Mice were intraperitoneally injected with LPS (12.5 mg/kg) 2 h after the intraperitoneal injection of 0.5 mL ozone (15 μg/mL). A: The content of cf-DNA in blood was measured at 12 h and 24 h after the injection of LPS using the Quant-iT PicoGreen dsDNA Assay kit ( *n* = 6). B: Representative confocal immunofluorescence microscopy images of the lung tissues from the mice treated with LPS for 12 h and stained with H3Cit (green) and DNA (blue). Scale bar, 50 μm. C–E: The protein levels of TF, MMP-9, and IL-1β in the lung tissues were detected by Western blotting at 12 h after the initiation of LPS treatment ( *n* = 6). Data are presented as mean ± standard deviation. ^*^ *P* < 0.05, ^**^ *P* < 0.01, and ^***^ *P* < 0.001 by one-way ANOVA followed by Tukey's tests for multiple comparisons. Abbreviations: LPS, lipopolysaccharide; H3Cit, citrullinated histone H3; TF, tissue factor; MMP-9, matrix metalloproteinase-9; IL-1β, interleukin-1β.

As previously mentioned, neutrophils constitute a significant proportion of infiltrated inflammatory cells in the lung tissues, and it was reported that neutrophil-derived NETs promoted the progression of lung injury ^[ 14] ^. Therefore, we examined the level of cf-DNA, which partially represents the residue of NETs in plasma. Following the LPS injection, cf-DNA levels remained elevated during the 12- to 24-h time frame. However, in the ozone-pretreated mice, the concentration of cf-DNA in plasma was significantly lower than that of the model group, and by 24 h after LPS injection, the cf-DNA level was almost restored to physiological levels ( ***Fig. 2A***). Furthermore, confocal microscopy showed that LPS increased the fluorescence intensity of H3Cit, which might be decreased by ozone pretreatment, in the lungs of mice ( ***Fig. 2B***). Therefore, it is reasonable to suspect that ozone may reduce the abnormal accumulation of NETs.

Clinical practices show that the majority of patients with sepsis have coagulation abnormalities ^[ 31– 33] ^. TF plays a key role in initiating the coagulation cascade, and excessive release of pathological TF by monocytes/macrophages during sepsis may lead to thrombosis. Therefore, we evaluated TF expression. As depicted in ***Fig. 2C***, LPS administration increased TF expression in lung tissue, while ozone pretreatment resulted in a reduction of TF expression. Given the presence of red blood cell leakage observed in the histological results ( ***Fig. 1C***), we presumed an elevation in matrix metalloproteinase (MMP) expression, which was indeed confirmed ( ***Fig. 2D***). Additionally, the expression of IL-1β, an indicator of inflammation development, also increased ( ***Fig. 2E***). Both the MMP expression and IL-1β levels, which might contribute to lung injury exacerbation, were inhibited by ozone treatment ( ***Fig. 2D*** and ***2E***).

### Ozone ameliorated lung injury by inhibiting NETs

**Figure 3:**
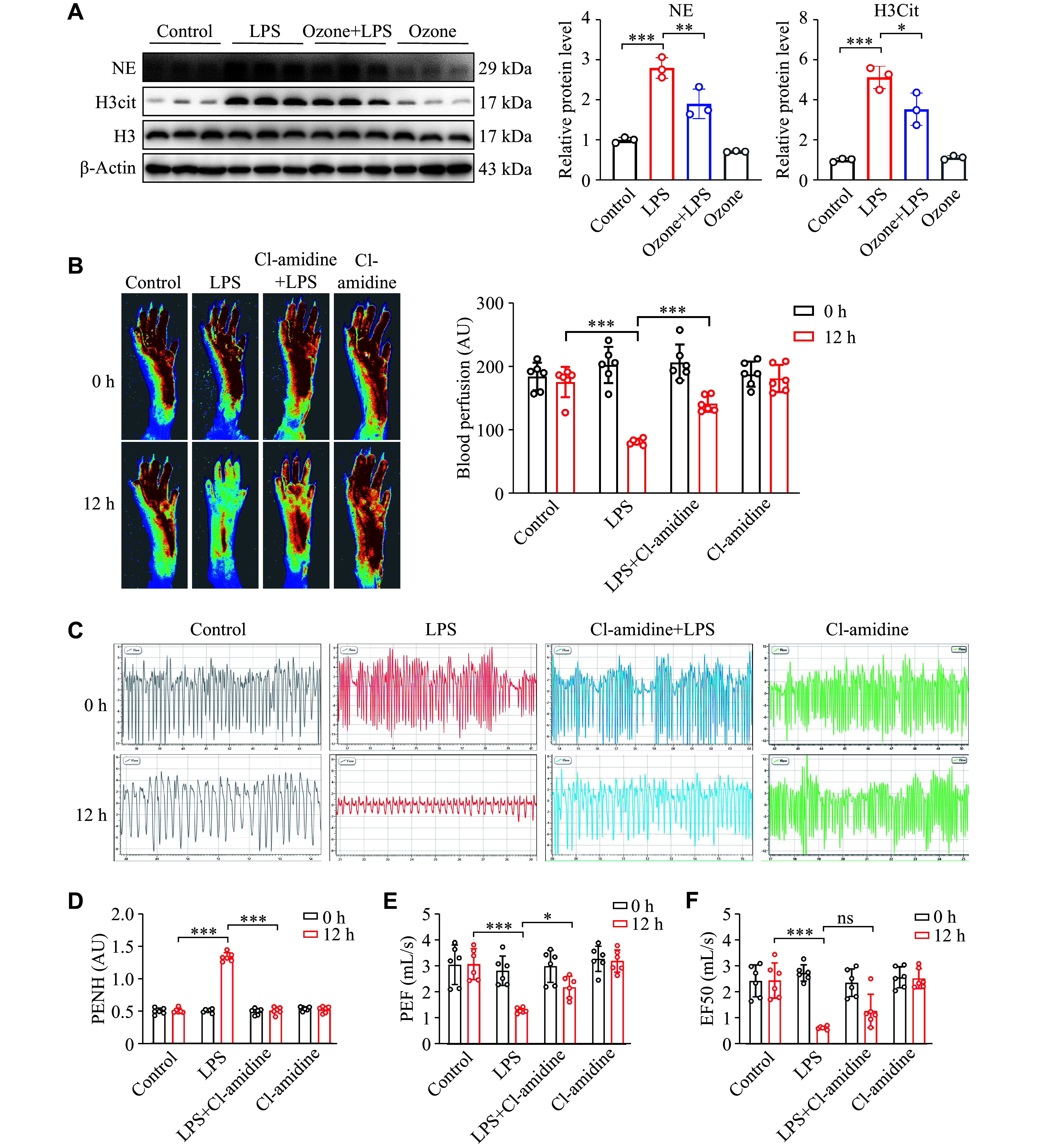
Ozone inhibiting the LPS-induced accumulation of NETs. A: Neutrophils were isolated from the bone marrow of mice using Histopaque 1077 and 1119. The NET formation was induced by stimulating neutrophils with LPS (1 μg/mL) for 12 h after treating the neutrophils with ozone (15 μg/mL) for 2 h. The protein levels of H3Cit and NE in neutrophils were detected by Western blotting ( *n* = 3). B–F: Mice were injected intraperitoneally with LPS (12.5 mg/kg) 2 h after the intraperitoneal injection of Cl-amidine (10 mg/kg). Blood flow in the lower limbs of mice was evaluated at 0 h and 12 h after the LPS injection ( *n* = 6; B). Representative eight-second waveforms of lung function at 0 h and 12 h after the LPS injection (C). Indexes of the lung function include enhanced pause (PENH), peak expiratory flow (PEF), and mid-expiratory flow (EF50) at 0 h and 12 h after the LPS injection ( *n* = 6; D–F). Data are presented as mean ± standard deviation. ^*^ *P* < 0.05, ^**^ *P* < 0.01, and ^***^ *P* < 0.001 by one-way ANOVA followed by Tukey's tests for multiple comparisons. Abbreviations: LPS, lipopolysaccharide; NE, neutrophil elastase; H3Cit, citrullinated histone H3; AU, arbitrary unit; ns, not significant.

To test the hypothesis that ozone inhibited NETs, we administered ozone before LPS induced the formation of NETs in neutrophils. The results showed that ozone was able to reduce the protein levels of H3Cit and NE, demonstrating that ozone has the properties that inhibit the generation of NETs ( ***Fig. 3A***). As is known, the bacteria-induced NET formation is mediated by PAD4 in ALI ^[ 34] ^. Therefore, we used a PAD4 inhibitor, Cl-amidine, to mitigate sepsis-induced lung injury by reducing NET formation. The results showed that Cl-amidine treatment significantly reversed LPS-induced microcirculation disturbance in the extremities ( ***Fig. 3B***). Furthermore, Cl-amidine significantly improved the lung function of mice induced by LPS, and restored several important parameters to normal levels ( ***Fig. 3C**– **3F***). These findings provided evidence that ozone therapy targeting the reduction of NETs represented a potential therapeutic strategy for treating ALI.

### The *Sr-a1* gene knockout abolished the effect of ozone on lung injury

**Figure 4:**
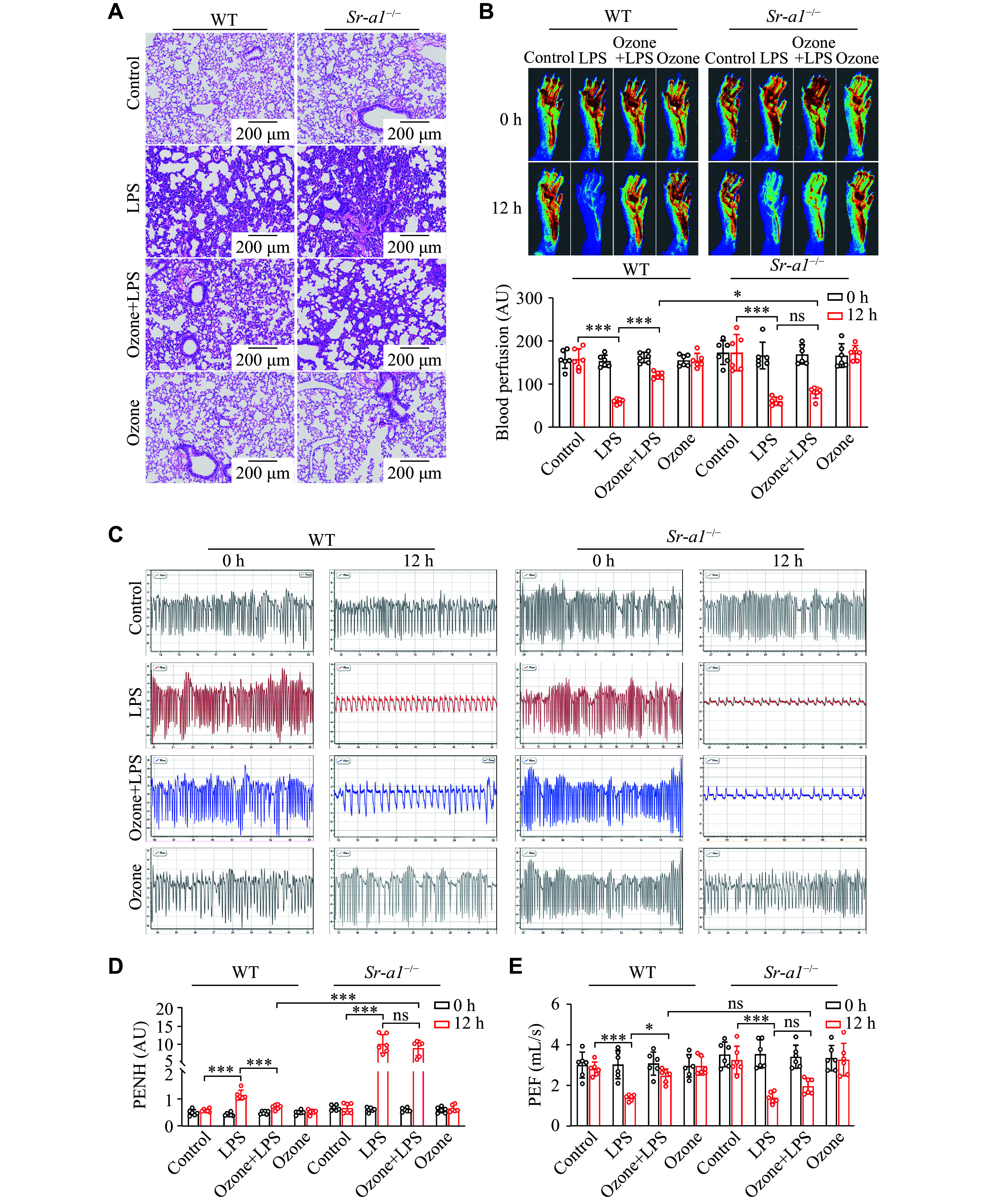
Knockout of the *Sr-a1* gene abolished the effect of ozone on the LPS-induced lung injury. Mice were injected intraperitoneally with LPS (12.5 mg/kg) 2 h after the intraperitoneal injection of 0.5 mL ozone (15 μg/mL). A: H&E sections of the lung tissues after injection of LPS for 12 h ( *n* = 3). Scale bar, 200 μm. B: Blood flow in the lower limbs of mice was evaluated at 0 h and 12 h after the LPS injection ( *n* = 6). C: Representative eight-second waveforms of lung function at 0 h and 12 h after the LPS injection. D and E: Indexes of lung function include enhanced pause (PENH) and peak expiratory flow (PEF) ( *n* = 6). Data are presented as mean ± standard deviation. ^*^ *P* < 0.05 and ^***^ *P* < 0.001 by two-way ANOVA followed by Tukey's tests for multiple comparisons. Abbreviations: LPS, lipopolysaccharide; AU, arbitrary unit; ns, not significant.

It has been reported that the scavenger receptor family mediates the clearance of apoptotic neutrophils by resident alveolar macrophages ^[ 35] ^. Therefore, it is reasonable to assume that the scavenger receptor plays a key role in the removal of NETs. Compared with the wild-type (WT) mice, lung damage in the *Sr-a1* ^*−/−*^ mice did not decrease, even with ozone treatment. Specifically, the H&E staining showed that the lung focal consolidation of the *Sr-a1* ^*−/−*^ mice was more severe after LPS injection. Even after ozone treatment, the lung structure remained damaged, with a significant presence of red blood cells and infiltrated inflammatory cells ( ***Fig. 4A***). Furthermore, ozone failed to increase average blood flow in the lower limbs of the *Sr-a1* ^*−/−*^ mice, further suggesting the essential role of SR-A1 in the protective effects of ozone ( ***Fig. 4B***). Consistent with previous findings, in the current study, the protective effect of ozone was observed to be absent in mice with the knocked-out *Sr-a1* gene. As depicted in ***Fig. 4C***, following a 12-hour stimulation with LPS in the *Sr-a1* ^*−/−*^ mice, there was a significant decrease in respiratory frequency and tidal volume, and the respiratory function was not significantly restored, even with the early administration of ozone. Further parametric analysis revealed that the *Sr-a1* ^*−/−*^ mice had a 20-fold increase in the enhanced pause (PENH), compared with that of the WT mice after LPS treatment, reflecting a severe deterioration in their airway conductivity ( ***Fig. 4D*** and ***4E***). The therapeutic effects of ozone in WT mice were not replicated in the *Sr-a1* ^*−/−*^ mice, highlighting the essential role of SR-A1 in mediating the protective effects of ozone.

### The *Sr-a1* gene knockout abolished ozone-inhibited NETosis

**Figure 5:**
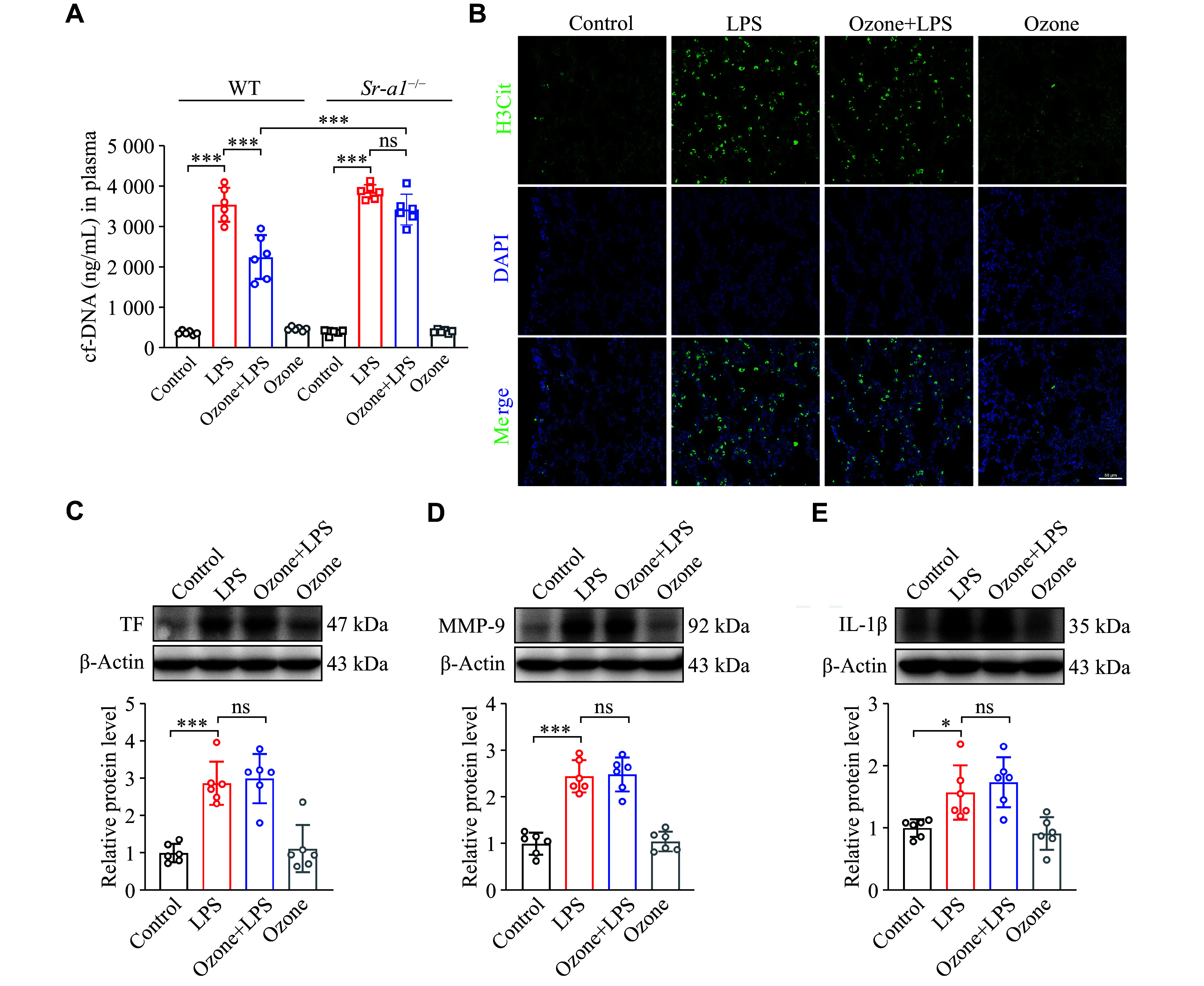
Knockout of the *Sr-a1* gene abolished the inhibition of ozone on NETs. Mice were injected intraperitoneally with LPS (12.5 mg/kg) 2 h after the intraperitoneal injection of 0.5 mL ozone (15 μg/mL). A: The content of cf-DNA in blood was measured at 12 h after the injection of LPS using the Quant-iT PicoGreen dsDNA Assay kit ( *n* = 6). B: Representative confocal immunofluorescence microscopy images of the lung tissues from the *Sr-a1* ^*−/−*^ mice treated with LPS for 12 h and stained with H3Cit (green) and DNA (blue) ( *n* = 3), scale bars: 50 μm. C–E: The protein levels of TF, MMP-9, and IL-1β in the *Sr-a1* ^*−/−*^ mouse lung tissues were detected by Western blotting at 12 h after the initiation of LPS treatment ( *n* = 6). Data are presented as mean ± standard deviation. ^*^ *P* < 0.05 and ^***^ *P* < 0.001 by one-way ANOVA followed by Tukey's tests for multiple comparisons. Abbreviations: LPS, lipopolysaccharide; H3Cit, citrullinated histone H3; TF, tissue factor; MMP-9, matrix metalloproteinase-9; IL-1β, interleukin-1β; ns, not significant.

In the current study, we assessed the levels of cf-DNA in the plasma of the *Sr-a1* ^*−/−*^ mice. Consistent with the WT mice after LPS stimulus, the levels of cf-DNA were elevated, while ozone failed to reduce the cf-DNA content in the *Sr-a1* ^*−/−*^ mice ( ***Fig. 5A***). The confocal microscopy analysis showed that LPS induced stronger fluorescence intensity of H3Cit in the lungs of the *Sr-a1* ^*−/−*^ mice, which was only slightly attenuated by ozone pretreatment ( ***Fig. 5B***). The increased expression levels of TF, IL-1β, and MMP-9 induced by LPS in the *Sr-a1* ^*−/−*^ mice were not significantly blocked by ozone pretreatment ( ***Fig. 5C***– ***5E***). These findings suggested that in the absence of the SR-A1, ozone failed to exert its effects, indicating the involvement of SR-A1, at least partially, in the anti-inflammatory and lung-protective effects of ozone.

### The *Sr-a1* gene knockout abolished the ozone-mediated macrophage phagocytosis

**Figure 6:**
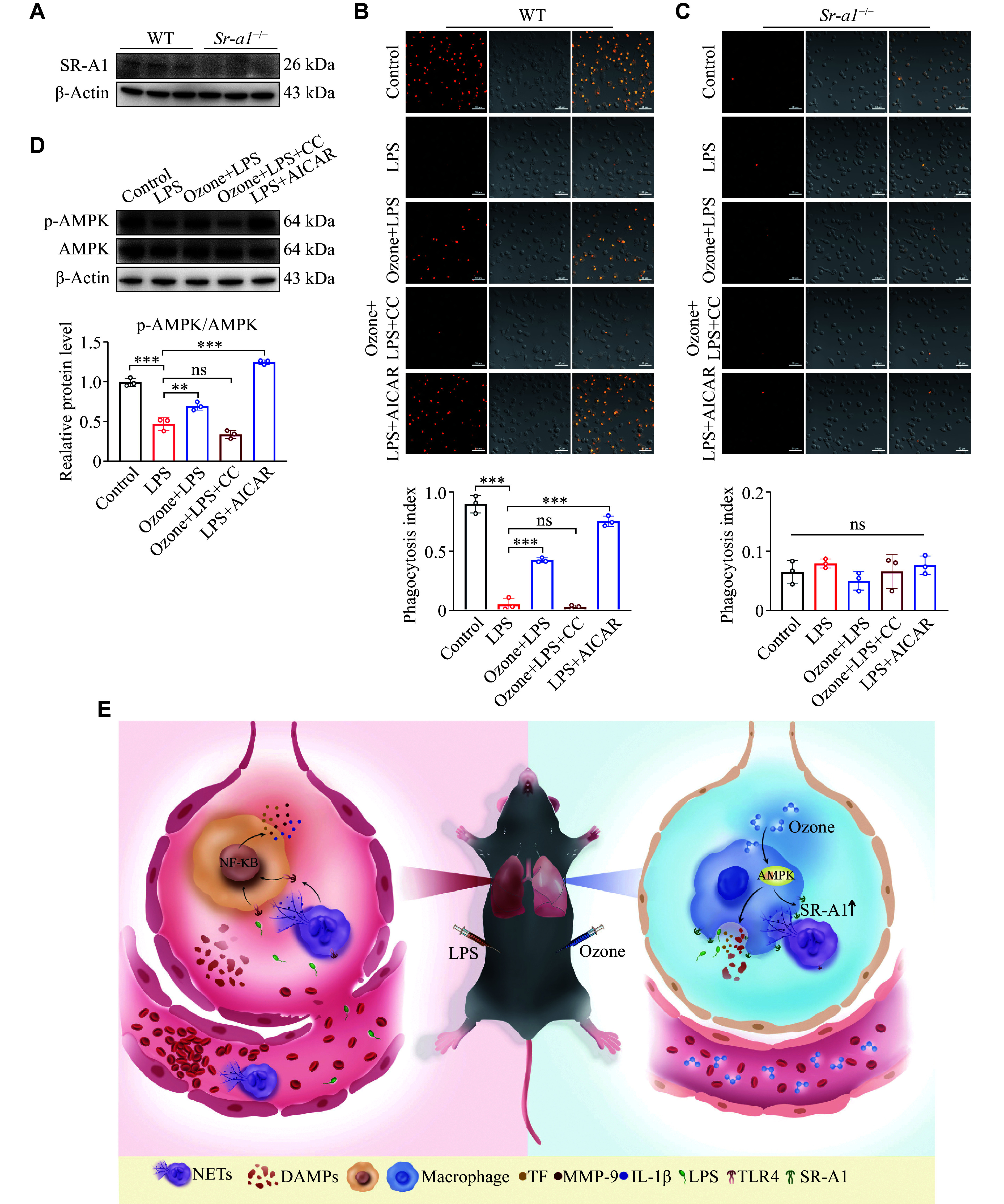
Knockout of the *Sr-a1* gene abolished the up-regulation of macrophage phagocytosis by ozone. A: The protein levels of SR-A1 of BMDMs from the wild-type (WT) mice or *Sr-a1* ^*−/−*^ mice were detected by Western blotting ( *n* = 3). B–D: BMDMs from the WT mice or *Sr-a1* ^*−/−*^ mice were induced to M1 type with LPS (100 ng/mL) and IFN-γ (50 ng/mL) for 24 h after differentiation and maturation *in vitro*. LPS (1 μg/mL) was used to stimulate BMDMs for 12 h to induce inflammation. Prior to this, BMDMs were pretreated with ozone (15 μg/mL) for 2 h, or with AICAR (300 μmol/L) or Compound C (20 μmol/L) for 15 min. Stained-labeled NETs (orange) were incubated with BMDMs processed as above in the confocal dish for 15 min, then rinsed lightly with ice-cold PBS three times, and photographed under confocal microscopy (B and C). NETs were labeled by Sytox Orange ( *n* = 3). Scale bar, 50 μm. The protein levels of p-AMPK/AMPK in BMDMs (from WT mice) were detected by Western blotting ( *n* = 3; D). E: The schematic illustration indicates that ozone enhances phagocytosis of NETs by macrophages through upregulation of SR-A1 *via* the AMPK pathway to alleviate septicemic lung injury. Data are presented as mean ± standard deviation. ^**^ *P* < 0.01 and ^***^ *P* < 0.001 by one-way ANOVA followed by Tukey's tests for multiple comparisons. Abbreviations: CC, Compound C; SR-A1, scavenger receptor A1; AMPK, AMP-activated protein kinase; NETs, neutrophil extracellular traps; DAMPs, damage-associated molecular patterns; TF, tissue factor; MMP-9, matrix metalloproteinase-9; IL-1β, interleukin-1β; LPS, lipopolysaccharide; TLR4, Toll-like receptor 4; ns, not significant.

Scavenger receptors are widely expressed in macrophages and are involved in pathogen recognition and clearance. First, we examined the SR-A1 protein levels in BMDMs, and found that SR-A1 was undetectable in BMDMs from the *Sr-a1* knockout mice ( ***Fig. 6A***). Then, we observed the macrophage phagocytosis in a live cell station. In BMDMs from the WT mice, almost all cells were capable of engulfing NETs, as indicated by the presence of orange particles within the BMDMs, while few NETs were found in BMDMs after LPS induction, indicating that LPS inhibited the phagocytosis of BMDMs. Upon the pretreatment of ozone, the phagocytic function of more than half of the BMDMs was restored. Notably, the upregulation of NET engulfment was abolished, when the AMPK inhibitor Compound C was combined with ozone treatment, while the AMPK agonist AICAR alone was able to counteract the inhibitory effect of LPS on macrophage phagocytosis ( ***Fig. 6B***). Conversely, BMDMs from the *Sr-a1* ^*−/−*^ mice were unable to engulf NETs regardless of any treatments ( ***Fig. 6C***). Meanwhile, the Western blotting results showed that ozone significantly promoted the phosphorylation of AMPK ( ***Fig. 6D***). Based on these findings, we hypothesized that ozone promoted the phagocytic function of macrophages by up-regulating the expression of SR-A1 through the AMPK pathway.

## Discussion

In the current study, we found that ozone administration resulted in an improved survival ratio and alleviated pulmonary edema in mice with LPS-induced sepsis. Furthermore, ozone treatment significantly reversed the decline in lung function, increased blood flow in the peripheral microcirculation, and ameliorated histopathological damage to the lung in the LPS-modeled mice. However, the therapeutic effects of ozone were nullified in mice by knocking out the SR-A1. Further studies showed that ozone treatment significantly inhibited the LPS-induced excessive accumulation of NETs and the protein levels of TF, MMP-9, and IL-1β *in vivo*, depending on the activation of macrophage SR-A1. These results suggest that ozone therapy may alleviate sepsis-induced ALI by activating the AMPK/SR-A1 axis to inhibit the abnormal accumulation of NETs.

Ozone has been recognized as a potent germicidal gas since the 19th century. Rather than acting as a substitute for antiviral drugs, ozone acts as a booster, exerting antiviral activity by inhibiting viral replication and directly inactivating viruses ^[ 36] ^. Combination therapy involving ozone and antiviral drugs has demonstrated the potential to reduce inflammation and lung damage ^[ 37] ^. Given these promising indications, we aimed to investigate the effect of ozone on the sepsis-induced ALI and explore its underlying mechanism. Ozone may be administered through various routes, including direct intravenous injection, major autohemotherapy, and extravascular blood oxygenation-ozonation. Since the effectiveness of ozone therapy depends on the gas concentration ^[ 38] ^, and ozone therapy is more effective in the early stages of viral diseases but has limited efficacy in severe or critical situations ^[ 37] ^, we established a concentration gradient of an ozone/oxygen mixture to determine the optimal dose for administration, with a pre-dose strategy.

Sepsis remains a prevalent, costly, and life-threatening condition worldwide. Because of its complex and dynamic nature, sepsis management requires a multifaceted approach ^[ 39] ^. Early-stage acute inflammation in sepsis is primarily mediated by neutrophils, while multiple inflammatory factors, such as tumor necrosis factor and various interleukins, may activate the coagulation cascade, leading to micro-thrombosis ^[ 40] ^. It has also been reported that in the initial stages of sepsis, the functional capillary ratio is significantly reduced because of the prolonged retention of neutrophils in small arteries and capillaries, ultimately resulting in pulmonary micro-circulatory disturbances and hypoxemia ^[ 41] ^. In the current study, histopathological analysis revealed an acute inflammatory response in the lungs characterized by significant neutrophil infiltration, which was substantially alleviated by ozone treatment, suggesting that ozone targets neutrophil infiltration and subsequent pulmonary micro-circulatory disturbances and hypoxemia.

Moreover, endotoxins induce microcirculatory disturbances, particularly in the liver and kidneys, thereby affecting hemodynamics during sepsis ^[ 42] ^. The impaired microcirculatory function is associated with the increased morbidity and mortality in various clinical scenarios ^[ 43] ^, and interventions aimed at improving microcirculation have shown organ-protective effects during sepsis ^[ 44] ^. In the current study, we assessed the lower extremity blood flow as an indicator, and the results demonstrated that LPS induction significantly reduced the lower extremity blood flow in mice, which was ameliorated by ozone therapy, further supporting the role of ozone in microcirculation improvement.

NETosis was originally recognized as a beneficial process wherein neutrophils released DNA as an active and specific form of host defense ^[ 45] ^. However, recent findings have indicated that NETs have a somewhat toxic nature. For example, patients with systemic lupus erythematosus or rheumatoid arthritis tended to produce NETs more readily, and components of NETs have been identified as pathogenic autoantigens, contributing to disease persistence and exacerbation ^[ 46– 47] ^. In addition, the formation of influenza-induced NETs in the lungs led to severe inflammation ^[ 48] ^. The current study sheds light on the negative implications of NETs. Ozone intervention demonstrated a significant inhibition of NET accumulation. To further determine the role of NETs in the ozone-treated ALI, we employed Cl-amidine (a chemical inhibitor of PAD) to inhibit the production of NETs, considering that PAD4 plays a crucial role in the histone citrullination step of NETosis ^[ 49] ^. Cl-amidine was able to mimic the therapeutic effect of ozone by improving lung function and enhancing lower limb circulation in the LPS-induced mice.

Clinical autopsy findings confirmed the presence of fibrin microthrombi in the lungs of patients with ARDS ^[ 50] ^. Recent studies have suggested that both the complement and TF-enriched NETs are crucial drivers of immune thrombosis in coronavirus disease 2019 (COVID-19) ^[ 51– 52] ^. Additionally, hirudin, an anticoagulant that targets endothelial cells, has effectively alleviated sepsis ^[ 53] ^. TF serves as a high-affinity receptor and cofactor for factor (F) Ⅶ/Ⅶ, playing a vital role in initiating coagulation and contributing to arterial and venous thrombosis ^[ 54] ^. Additionally, inflammatory stimuli may induce a coagulation state by triggering the TF production in both monocytes and endothelial cells ^[ 55] ^. MMPs are a class of metal ion-dependent endopeptidases responsible for degrading the extracellular matrix. While MMPs are produced by various cell types, recent studies have identified MMP-9, produced by neutrophils, as a significant factor in ALI. The inhibition of MMP-9 has been shown to attenuate the ventilator-induced lung injury in rats ^[ 56] ^. Moreover, the activated MMP-9 may cleave TFPI, disrupting the balance between TF and TFPI ^[ 57] ^. Collectively, the aforementioned studies demonstrated the essential roles of TF, MMP-9, and IL-1β in ALI, which aligns with the data obtained in the current study. We found that ozone significantly inhibited the LPS-induced protein expression of TF, MMP-9, and IL-1β in mouse lung tissues, leading to a reduction in pulmonary thrombus formation.

Low-density lipoprotein receptor-related protein 1 (LRP1), also known as scavenger receptor class L (SR-L), mediates the clearance of apoptotic neutrophils by resident alveolar macrophages, thereby suppressing the development of lung inflammation ^[ 35, 58] ^. Additionally, SR-L is involved in regulating matrix metalloproteinase activity to facilitate lung injury repair ^[ 35] ^. Interestingly, SR-L is responsible for the endocytosis and degradation of neutrophil elastase, which is necessary for the formation of NETs. Since NETs play a role in ALI, it is plausible to suggest that SR-L may have a beneficial effect in limiting the elastase-induced lung remodeling ^[ 59] ^.

As SR-A1 belongs to the SR family, we hypothesized that SR-A1 had a similar function to SR-L, specifically mediating macrophage clearance of NETs. The presence of SR-A6, predominantly expressed in macrophages, and its ability to clear bacteria from the blood and lungs further support our hypothesis. SR-A1 is capable of synergizing with Toll-like receptor 2 (TLR2) to enhance the phagocytosis of *Staphylococcus aureus* and with TLR4 to enhance the phagocytosis of *Escherichia coli*. The deficiency of SR-A1 in mice led to an increased mortality because of the *S. aureus* infection ^[ 18] ^.

To assess the involvement of SR-A1 in the sepsis-induced ALI, we used the *Sr-a1* ^*−/−*^ mice as an ALI model and examined the previously implicated indices. The results showed that after the *Sr-a1* knockout, the therapeutic efficacy of ozone was compromised, because the lung function and limb circulation disturbances were not restored by ozone, the accumulation of NETs increased, and the expression levels of TF, MMP9, and IL-1β proteins remained high, indicating that ozone exerted its effects through SR-A1. To further test our hypothesis that ozone enhanced macrophage phagocytosis *via* SR-A1, we extracted BMDMs from mice for *in vitro* experiments. Almost all BMDMs from the WT mice were able to phagocytose NETs, which disappeared after the LPS stimulation, and ozone partially restored this ability. However, BMDMs from the *SR-A1* ^*−/−*^ mice were unable to phagocytose NETs regardless of the ozone treatment. The above data provided some solid evidence of the role of SR-A1 in macrophage phagocytosis of NETs. Moreover, our recently published study demonstrated that activation of the AMPK/SR-A1 axis induced phagocytosis and degradation of HMGB1 by macrophages ^[ 60] ^. In line with this, the use of an AMPK activator and inhibitor allowed us to demonstrate that ozone upregulated SR-A1 through the AMPK signaling pathway, consequently enhancing macrophage phagocytosis of NETs. This finding aligns with the results obtained in our current study.

Most interstitial lung disease (ILD) is characterized by fibrosis or inflammation in the interstitial space, which leads to an impaired gas exchange and dyspnea ^[ 61] ^. Abnormally produced NETs have been documented as a possible trigger and exacerbator of ILD ^[ 62– 63] ^. *In vitro*, NETs have been shown to promote the activation and phenotypic differentiation of fibroblasts, and the percentage of fibrosis decreased after the degradation of NETs with DNase ^[ 64] ^. In a mouse model, neutrophil elastase inhibitors attenuated pulmonary fibrosis by inhibiting TGF-β activation and inflammatory cell recruitment in the lung ^[ 65] ^. PAD4 deficiency has also been proposed as a target for pulmonary fibrosis treatment ^[ 66] ^. Thus, we strongly recommend ozone therapy for the treatment of ILD, based on its ability to promote the clearance of NETs.

Indeed, it is important to recognize that the current study does not preclude the possibility of ozone exerting its therapeutic effects on ALI through alternative mechanisms. Ozone may directly act on Nrf2, whose activation inhibits iron overload and consequent oxidative stress induced by elevated levels of ferritin. Therefore, ozone's ability to prevent oxidative stress-induced apoptosis through this Nrf2-mediated pathway may contribute to its efficacy in ALI therapy ^[ 67– 68] ^. Exploring the activation of the antioxidant system represents a promising avenue for future investigations. It is worth noting that further clinical practice and investigation are still necessary to establish a consistently accepted protocol for the use of ozone. More studies are warranted to determine optimal dosages, administration routes, and potential adverse effects to ensure the safe and effective utilization of ozone in medical settings.

Collectively, our findings provide some compelling evidence that ozone treatment enhances the expression of macrophage SR-A1 through the AMPK pathway, leading to efficient clearance of excessive NETs and attenuation of septicemic lung injury. The reported incidence of sepsis is currently underestimated because of variations in definitions, triage processes, and reporting mechanisms in national guidelines. However, with the aging of the global population, the true incidence of sepsis is expected to gradually increase ^[ 7] ^. Ozone therapy, with its diverse positive effects, such as anti-inflammatory properties and immune enhancement, holds some promise as a novel immunotherapy. Combining ozone therapy with other drugs in patients with lung injury may yield reasonable, beneficial, and synergistic effects. Importantly, there are no known adverse or toxic effects associated with the appropriate use of ozone therapy. Ongoing research on the pathogenesis and treatment of sepsis will provide valuable insights at various levels, which is expected to improve sepsis prevention, awareness, and treatment strategies. Therefore, continued efforts in this field will contribute to advancing our understanding and management of sepsis.
